# Accuracy of diagnostic classification algorithms using cognitive-, electrophysiological-, and neuroanatomical data in antipsychotic-naïve schizophrenia patients

**DOI:** 10.1017/S0033291718003781

**Published:** 2018-12-18

**Authors:** Bjørn H. Ebdrup, Martin C. Axelsen, Nikolaj Bak, Birgitte Fagerlund, Bob Oranje, Jayachandra M. Raghava, Mette Ø. Nielsen, Egill Rostrup, Lars K. Hansen, Birte Y. Glenthøj

**Affiliations:** 1 Centre for Neuropsychiatric Schizophrenia Research & Centre for Clinical Intervention and Neuropsychiatric Schizophrenia Research, Mental Health Centre Glostrup, University of Copenhagen, Copenhagen, Denmark; 2 Faculty of Health and Medical Sciences, Department of Clinical Medicine, University of Copenhagen, Copenhagen, Denmark; 3 Cognitive Systems, DTU Compute, Department of Applied Mathematics and Computer Science, Technical University of Denmark, Kongens Lyngby, Denmark; 4 Department of Psychology, University of Copenhagen, Copenhagen, Denmark; 5 Department of Psychiatry, Brain Center Rudolf Magnus, University Medical Center Utrecht, Utrecht, The Netherlands; 6 Department of Clinical Physiology and Nuclear Medicine, Rigshospitalet, University of Copenhagen, Glostrup, Denmark

**Keywords:** Antipsychotic-naïve first-episode schizophrenia, cognition, diffusion tensor imaging, electrophysiology, machine learning, structural magnetic resonance imaging

## Abstract

**Background:**

A wealth of clinical studies have identified objective biomarkers, which separate schizophrenia patients from healthy controls on a group level, but current diagnostic systems solely include clinical symptoms. In this study, we investigate if machine learning algorithms on multimodal data can serve as a framework for clinical translation.

**Methods:**

Forty-six antipsychotic-naïve, first-episode schizophrenia patients and 58 controls underwent neurocognitive tests, electrophysiology, and magnetic resonance imaging (MRI). Patients underwent clinical assessments before and after 6 weeks of antipsychotic monotherapy with amisulpride. Nine configurations of different supervised machine learning algorithms were applied to first estimate the unimodal diagnostic accuracy, and next to estimate the multimodal diagnostic accuracy. Finally, we explored the predictability of symptom remission.

**Results:**

Cognitive data significantly classified patients from controls (accuracies = 60–69%; *p* values = 0.0001–0.009). Accuracies of electrophysiology, structural MRI, and diffusion tensor imaging did not exceed chance level. Multimodal analyses with cognition plus any combination of one or more of the remaining three modalities did not outperform cognition alone. None of the modalities predicted symptom remission.

**Conclusions:**

In this multivariate and multimodal study in antipsychotic-naïve patients, only cognition significantly discriminated patients from controls, and no modality appeared to predict short-term symptom remission. Overall, these findings add to the increasing call for cognition to be included in the definition of schizophrenia. To bring about the full potential of machine learning algorithms in first-episode, antipsychotic-naïve schizophrenia patients, careful *a priori* variable selection based on independent data as well as inclusion of other modalities may be required.

## Introduction

A wealth of clinical studies have successfully applied various objective measures to identify biomarkers, which separate schizophrenia patients from healthy controls on a group level. Although these studies have provided profound insight into the pathophysiology of schizophrenia, these efforts have not been translated into diagnostic utility (Kapur *et al*., 2012). Thus, the diagnosis of schizophrenia according to Diagnostic and Statistical Manual of Mental Disorder (DSM) and International Classification of Diseases (ICD) classifications entirely relies on clinical symptoms. Likewise, no clinical or objective measures for course of illness or response to antipsychotic medication have been implemented into clinical practice.

Numerous studies using objective cognitive test batteries such as Cambridge Neuropsychological Test Automated Battery (CANTAB) (Robbins *et al*., 1994) have established that cognitive deficits in, e.g. attention, verbal memory, and working memory are enduring and core features of schizophrenia, which are relatively unaffected by clinical state of the psychopathological symptoms (Paulus *et al*., 2001; Gur *et al*., 2006; Kahn and Keefe, 2013).

Assessment of early information processing as measured with electrophysiological paradigms has also indicated impairments in schizophrenia patients, and also these disturbances are generally considered unaffected by disease stage and severity of symptoms (Koychev *et al*., 2012; Thibaut *et al*., 2015; Blakey *et al*., 2018). Commonly used electrophysiological paradigms comprise P50 suppression (Adler *et al*., 1982), pre-pulse inhibition of the startle response (PPI) (Braff and Geyer, 1990), and mismatch negativity (MMN) (Shelley *et al*., 1991).

Finally, magnetic resonance imaging (MRI) has demonstrated that schizophrenia is associated with structural brain changes (Haijma *et al*., 2013). Gray matter structures have commonly been assessed with a region of interest (ROI) approach, but the development of diffusion tensor imaging (DTI) techniques such as tract-based spatial statistics have enabled assessment of the cerebral white matter microstructure (Smith *et al*., 2006). Overall, both subtle gray (Shepherd *et al*., 2012; Gong *et al*., 2016) and white matter (Fitzsimmons *et al*., 2013; Canu *et al*., 2015) deficits are present already at illness onset and before initiation of antipsychotic medication.

From a clinical perspective, the current categorical diagnostic systems contrast the multifaceted clinical phenotype of schizophrenia, and it is plausible that schizophrenia is better conceptualized using a more dimensional view (Jablensky, 2016). The research domain criteria (RDoC) were formulated to conceptualize integration of data ranging from basic biological levels to behavioral constructs across mental disorders (Insel *et al*., 2010). Theoretically, subgroups of schizophrenia patients may share certain pathophysiological disturbances, which can serve as targets for treatment with enhanced precision (Bak *et al*., 2017). In order to operationalize the RDoC approach, novel analysis strategies, which are sensitive to subtle signals in rich datasets, may be advantageous.

Categorical separation of groups is classically investigated with application of univariate statistical tests on unimodal data. It is increasingly appreciated that application of advanced multivariate, supervised machine learning algorithms on multimodal data may provide an improved framework for operationalizing the complex, dimensional clinical characteristics in, e.g. schizophrenia (Veronese *et al*., 2013; Dazzan, 2014). In short, a supervised machine learning algorithm identifies ‘patterns’ in complex data, which are not modelled by more classical statistical methods. Next, these patterns can be used to predict the outcome (e.g. ‘schizophrenia’ *v.* ‘healthy’; or ‘remission’ *v.* ‘non-remission’) for future, independent, individual observations with an estimated ‘accuracy’. Various algorithms have been developed, each with their own advantages and disadvantages depending on, e.g. the variance and distribution of the data (Bishop, 2006; Cawley and Talbot, 2010). Previous machine learning studies have generated encouraging diagnostic accuracies >85% (e.g. Shen *et al*., 2014; Chu *et al*., 2016; Santos-Mayo *et al*., 2017; Xiao *et al*., 2017) as well as prediction of the clinical outcome (Zarogianni *et al*., 2017). However, most previous studies have been unimodal and performed in medicated and more chronic patient samples, in which the variation in data is greater than at first illness presentation. Studies investigating multiple modalities in antipsychotic-naïve schizophrenia patients are absent.

In this proof-of-concept study, we applied nine configurations of different supervised machine learning algorithms, and we first compared the diagnostic accuracies of cognition, electrophysiology, structural MRI (sMRI), and DTI in a sample of first-episode, antipsychotic-naïve schizophrenia patients and healthy controls. Tests of group differences were supplemented with univariate analyses. Next, we investigated if combinations of modalities improved the diagnostic accuracy. Finally, we explored the predictive accuracy with regard to symptom remission after 6 weeks of antipsychotic monotherapy with amisulpride. We hypothesized that all four modalities would significantly discriminate patients from controls, and we expected higher accuracies for multimodal analyses.

## Materials and methods

### Trial approval

The authors assert that all procedures contributing to this work comply with the ethical standards of the Danish National Committee on Biomedical Research Ethics (H-D-2008-088) and with the Helsinki Declaration of 1975, as revised in 2008. All participants approved participation by signing informed consent. Clinical trials identifier: NCT01154829.

### Participants

As part of a comprehensive multimodal study conducted between December 2008 and 2013, we recruited antipsychotic-naïve first-episode schizophrenia patients from psychiatric hospitals and outpatient mental health centers in the Capital Region of Denmark. Unimodal data on electrophysiology (Düring *et al*., 2014, 2015), DTI (Ebdrup *et al*., 2016), global cortical structures (Jessen *et al*., 2018), as well as data on cognition in combination with electrophysiology (Bak *et al*., 2017) have previously been published.

Patients were aged 18–45 years and all were lifetime naïve to any antipsychotic or methylphenidate exposure. Patients underwent a structured diagnostic interview (Schedule of Clinical Assessment in Neuropsychiatry, SCAN, version 2.1) to ensure fulfilment of ICD-10 diagnostic criteria of schizophrenia or schizoaffective psychosis (Wing *et al*., 1990). Inclusion required a normal physical and neurological examination and no history of major head injury. Previous diagnoses of drug dependency according to ICD as well as current recreational drug use were accepted. A current diagnosis of drug dependency was an exclusion criterion. Current drug status was measured by urine test (Rapid Response, Jepsen HealthCare, Tune, Denmark). Patients treated with antidepressant medication within the last month or during the study period were excluded. Benzodiazepines and sleep medication were allowed until 12 h prior to examination days.

Duration of untreated illness (DUI) was defined as the period in which the patient reported a continuous deterioration of functioning due to disease-related symptoms (Crespo-Facorro *et al*., 2007). Level of function was assessed with the Global Assessment of Function (GAF) and the Clinical Global Impression Scale (CGI) (Busner and Targum, 2007). Symptom severity was assessed by trained raters using the Positive and Negative Syndrome Scale (PANSS) (Kay *et al*., 1987). After completing all baseline examinations, patients commenced amisulpride monotherapy for 6 weeks. Dosing of amisulpride was adjusted aiming to optimize clinical effect and minimize side effects. Use of anticholinergic medication was not allowed. Symptom remission after 6 weeks was assessed using the Andreasen criteria (Andreasen *et al*., 2005).

Healthy controls matched on age, gender, and parental socioeconomic status were recruited from the community. Controls were assessed with a SCAN interview, and former or present psychiatric illness, substance abuse, or first-degree relatives with psychiatric diagnoses, were exclusion criteria. Demographic data are presented in Table 1.

**Table 1.** Demographical and clinical data. Lifetime use of tobacco, alcohol, cannabis, stimulants, hallucinogens, and opioids were categorized according to an ordinal five-item (0 = never tried/1 = tried few times/2 = use regularly/3 = harmful use/4 = dependency)

	Schizophrenia		Healthy controls		
	*N*	Mean (SD)	*N*	Mean (SD)	*p*
Age, years	46	25.0 (5.6)	58	24.79(5.68)	0.79^a^
Gender (m/f)	46	28/18	58	36/22	0.901^b^
Parental SES (a/b/c)	44	8/30/6	56	16/30/10	<0.001^b^
Years of education	45	12.5 (2.6)	56	14.58(2.60)	<0.001^a^
Danish Adult Reading Test (DART)^c^	41	21.4 (9.7)	56	23.2 (6.3)	0.27^d^
Total IQ (WAIS III)^e^	41	−0.8 (1.5)	54	0.0 (1.0)	0.002^d^
Tobacco (0/1/2/3/4)	45	7/11/22/1/4	59	13/29/11/2/1	0.003^f^
Alcohol (0/1/2/3/4)	46	2/6/33/4/1	57	3/1/53/0/0	0.005^f^
Cannabis (0/1/2/3/4)	46	8/23/9/6/0	57	23/28/6/0/0	0.003^f^
Opioids (0/1/2/3/4)	46	38/8/0/0/0	56	52/4/0/0/0	0.132^f^
Stimulants (0/1/2/3/4)	46	27/14/5/0/0	56	47/9/0/0/0	0.003^f^
Hallucinogens (0/1/2/3/4)	45	38/7/0/0/0	57	53/3/0/0/0	0.105^f^
Other drugs (0/1/2/3/4)	43	40/3/0/0/0	56	54/2/0/0/0	0.65^f^
Benzodiazepines (0/1/2/3/4)	42	31/11/0/6/0	55	55/0/0/0/0	<0.001^f^
DUI, weeks	45	65.8 (70.5)	–	–	–
CGI, severity	44	4.2 (0.7)	–	–	–
GAF, symptom	44	40.9 (9.9)	–	–	–
GAF, function	43	42.6 (11.1)	–	–	–
PANSS, positive	46	20.1 (4.2)	–	–	–
PANSS, negative	46	21.3 (7.9)	–	–	–
PANSS, general	46	42.2 (9.4)	–	–	–
PANSS, total	46	83.5 (17.2)	–	–	–
Amisulpride, mg/day	32	248.4 (140.6)	–	–	–
Remission (yes/no)^g^	34	11/23	–	–	–

SES, parental socioeconomic status; DUI, duration of untreated illness; CGI, Clinical Global Impression Scale; GAF, Global Assessment of Functioning; PANSS, Positive And Negative Syndrome Scale.

a Mann–Whitney *U* test.

b χ^2^.

c Danish Adult Reading Test (DART) (Nelson and O'Connell, 1978).

d Two-sample *t* test with pooled variance estimates.

e A combined score based on four subtests from WAIS III: Wechsler Adult Intelligence Scale (Wechsler Adult Intelligence Scale^®^ – Third Edition n.d.), presented as *z*-scores standardized from the mean and standard deviation of the healthy control sample.

^f^Fisher's exact test.

^g^Symptom remission after 6 weeks according to Andreasen criteria (Andreasen *et al*., 2005).

### Cognition

A comprehensive neurocognitive test battery was used to assess all participants, administered by research staff trained and supervised in the standardized administration and scoring of the battery. We included variables from the following neurocognitive tasks: Danish Adult Reading Test (DART) (Nelson and O'Connell, 1978), Wechsler Adult Intelligence Scale (WAIS III) (Wechsler Adult Intelligence Scale^®^ – Third Edition n.d.), Brief Assessment of Cognition in Schizophrenia (BACS) (Keefe *et al*., 2004), and Cambridge Neuropsychological Test Automated Battery (CANTAB) (Robbins *et al*., 1994), yielding a total of 25 cognitive variables for the current study [listed in online Supplementary Material (Table S1)].

### Electrophysiology

The Copenhagen Psychophysiology Test Battery (CPTB) was used to examine all participants (Düring *et al*., 2014, 2015). Auditory stimuli were presented by a computer running ‘Presentation’ (Neurobehavioral Systems, Inc., Albany, NY, USA) software (soundcard: Creative soundblaster 5.1, 2008 Creative Technology Ltd, Singapore, Singapore). Stimuli were presented binaurally through stereo insert earphones (Eartone ABR, 1996–2008 Interacoustics A/S, Assens, Denmark; and C and H Distributors Inc, Milwaukee, WI, USA). To avoid cross-test influences, the CPTB is always assessed in a fixed order, including PPI, P50 suppression, MMN, and selective attention paradigms, yielding a total of 19 electrophysiological variables for the current study [listed in online Supplementary Material (Table S1)].

### Neuroanatomy

MRI scans were acquired with a Philips Achieva 3.0 T whole body MRI scanner (Philips Healthcare, Best, The Netherlands) with an eight-channel SENSE Head Coil (Invivo, Orlando, Florida, USA).

### Structural MRI

The three-dimensional high-resolution T1-weighted images (repetition time 10 ms, echo time 4.6 ms, flip angle 8°, voxel size 0.79 × 0.79 × 0.80 mm) were acquired and processed through FSL pipelines (Jenkinson *et al*., 2012) comprising the following steps: (1) brain extraction; (2) brain segmentation using the ‘fslanat’ algorithm, and resulting in gray and white matter partial volume maps for each subject; (3) non-linear warping of structural images to MNI standard space, and subsequent application of the transformation matrices to the tissue maps; (4) modulation of the warped maps using the Jacobian determinant in order to maintain local gray matter volume during the non-linear warping. Finally, regional gray matter volumes were extracted from each of the 48 anatomical regions per hemisphere derived from the Harvard–Oxford cortical atlas as specified by FSL. The total brain volume and relative ventricular volume were determined using the FSL-SIENAX program. For the brain structural analyses, we *a priori* applied the ROI approach since ROI analyses have been widely applied in the field (Haijma *et al*., 2013), and we aimed to optimize the external validity and reproducibility of the results. These procedures yielded a total of 98 sMRI variables for the current study [listed in online Supplementary Material (Table S1)].

### Diffusion tensor imaging

Whole brain DTI images were acquired using single-shot spin-echo echo-planar imaging and a total of 31 different diffusion encodings [five diffusion unweighted (*b* = 0 s/mm^2^) and 30 diffusion weighted (*b* = 1000 s/mm^2^) non-collinear directions]. Acquired matrix size = 128 × 128 × 75; voxel dimensions = 1.88 × 1.88 mm × 2 (no slice gap); TR/TE = 7035/ 68 ms; flip angle = 90°. Images were processing using the FSL library of tools (Jenkinson *et al*., 2012). Diffusion parameter maps of fractional anisotropy (FA), mean diffusivity (MD), parallel diffusivity (*λ*1), radial diffusivity (*λ*23) and mode of anisotropy (MO) were derived using DTIFIT as previously described (Ebdrup *et al*., 2016). The mean values of these five diffusion parameters were extracted from 20 regions (based on the JHU white matter tractography atlas) and yielded a total of 100 DTI variables for the current study [listed in online Supplementary Material (Table S1)].

### Statistical methods

Statistical Package for the Social Sciences software (version 22, SPSS Inc., USA) was used to analyze demographic and clinical data. The distribution of continuous data was tested for normality with the Shapiro–Wilk test. Data on age and years of education were not normally distributed, and group comparisons were performed non-parametrically with the Mann–Whitney U test. Group differences in gender and socioeconomic status were tested with Pearson's χ^2^ test, and differences in abuse variables were tested with Fisher's exact test. Group differences in DART and estimated total IQ from WAIS III were tested using two-sample *t* tests with pooled variance estimates in MATLAB^®^.

### Machine learning algorithms

**Fig. 1.**
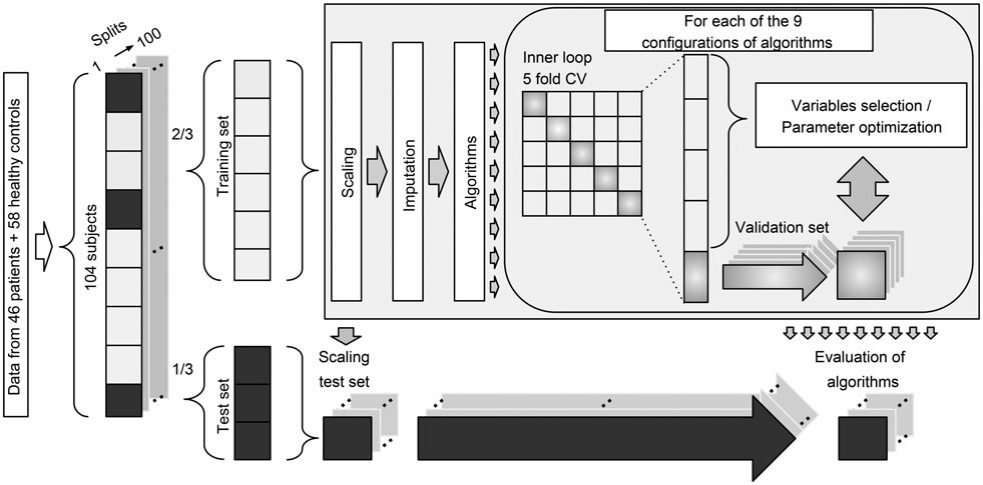
Diagram of the multivariate analysis pipeline. Forty-six patients and 58 healthy controls were included in the baseline analyses. ‘Data’ refer to input variables from cognition, electrophysiology, structural magnetic resonance imaging, and diffusion tensor imaging. For each of the 100 splits, 2/3 of subjects were used for training and 1/3 of subjects were used for testing. Subjects with missing data were not used in test sets. Training data were scaled (zero mean, unit variance), and the test sets were scaled using these parameters. Missing data were imputed using *K*-nearest neighbor imputation with *K* = 3 (Bak and Hansen, 2016), and only subjects with complete data were included in the test sets. Finally, nine different configurations of machine learning algorithms were applied to predict diagnosis. CV = cross-validation. See text for details.

We included participants with available data from all four modalities. We allowed subjects to have missing data points in up to 12 variables across all modalities. Twelve patients and 13 healthy controls had missing variables in the cognitive and electrophysiological data. Missing data were imputed as part of the analysis pipeline using *K*-nearest neighbor imputation with *K* = 3 (Bak and Hansen, 2016). Imputation of missing data was performed as part of the 100 random subsamples cross-validation (CV) loop, and thus the imputation procedure was only performed within the training set of a given split. We used a total of nine different configurations involving six machine learning algorithms: naïve Bayes (nB), logistic regression, support vector machine (SVM) (Cortes, 1995), decision tree (DT) (Breiman *et al*., 1984), random forest (RF) (Breiman, 2001), and auto-sklearn (AS) (Feurer *et al*., 2015). The algorithms were selected *a priori* based on their common usage and their proposed strength in relatively small datasets. To ensure comparability across all algorithms and modalities, the same pipeline and set-up were used for all analyses (Fig. 1).

### Analysis pipeline

To estimate the generalization error, we used random subsampling CV (Varoquaux *et al*., 2017) with 100 stratified splits of patients and controls (Fig. 1). This approach ensured that all configurations of algorithms were trained on the same data, and the ratio between the two classes was similar for all splits. Therefore, the performance of algorithms was evaluated on the same test data. For each split, one-third of the data was used for testing and two-thirds were used for training. All data imputation, feature selection, model training, and optimization were based exclusively on the training set of a given split. Logistic regression was used in two configurations: with L1 regularization (LR_r) and without regularization (LR). SVM was used in three configurations: one with a linear kernel (SVM_l), one with a radial basis function kernel using heuristic parameters (SVM_h), and one with optimized parameters (SVM_o). An inner loop fivefold CV was used to optimize model parameters (LR_r, SVM_o) or perform backwards elimination feature selection (LR, SVM_l, SVM_h, DT). Algorithms RF and AS have inherent parameter optimization, and therefore these configurations required no inner loop CV. See online Supplementary Material ‘Machine learning algorithms’ for details.

### Strategy for analyses

**Fig. 2.**
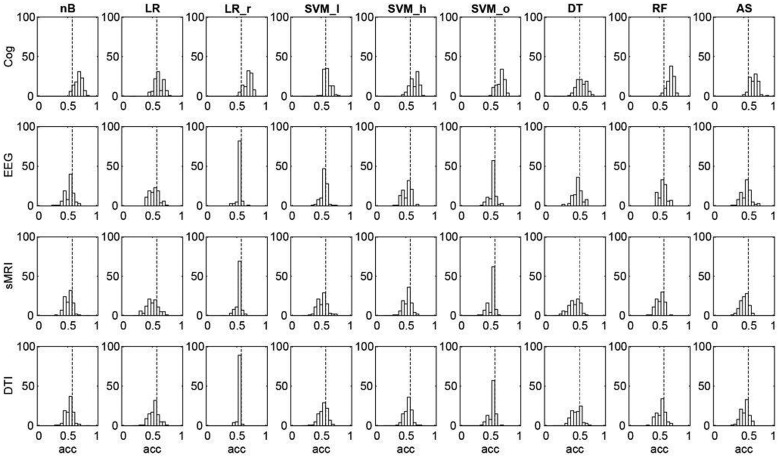
Unimodal diagnostic accuracies for cognition (Cog), electrophysiology (EEG), structural magnetic resonance imaging (sMRI), and diffusion tensor imaging (DTI) for each of the nine different configurations of machine learning algorithms. *X*-axes show the accuracies (acc), and *y*-axes show the sum of correct classifications for each of the 100 random subsamples (see Fig. 1). Dotted vertical black line indicates chance accuracy (56%). With cognitive data, all nine configurations of algorithms significantly classified ‘patient *v.* control’ (*p* values = 0.001–0.009). No algorithms using EEG, sMRI, and DTI-data resulted in accuracies exceeding chance. The nine different configuration of machine learning algorithms: nB, naïve Bayes; LR, logistic regression without regularization; LR_r, logistic regression with regularization; SVM_l, support vector machine with linear kernel; SVM_h, SVM with heuristic parameters; SVM_o, SVM optimized through cross-validation; DT, decision tree; RF, random forest; AS, auto-sklearn. See text for details.

**Fig. 3.**
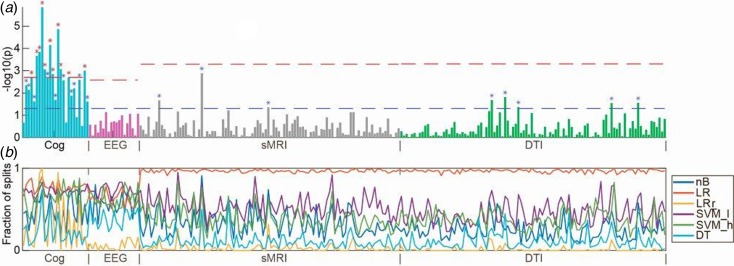
(*a*) Manhattan plot with univariate *t* tests of all variables along the *x*-axis [cognition (Cog), electrophysiology (EEG), structural magnetic resonance imaging (sMRI), and diffusion tensor imaging (DTI)] and log-transformed *p* values along the *y*-axis. Lower dashed horizontal line indicates significance level of *p* = 0.05. Upper dashed lines indicate the Bonferroni-corrected *p* value for each modality. (*b*) In colored horizontal lines, the fraction of data splits (see Fig. 1), where individual variables were included in the final machine learning model, which determined the diagnostic accuracy (presented in Fig. 2). Specification of variables is provided in online Supplementary Material. Only configurations of the six machine learning algorithms, which included feature selection, are shown. nB, naïve Bayes; LR, logistic regression without regularization; LR_r, logistic regression with regularization; SVM_l, support vector machine with linear kernel; DT, decision tree; RF, random forest.

To acquire unimodal estimates for the ability to separate patients from healthy controls (i.e. the ‘diagnostic accuracy’), data from each of the four modalities (cognition, electrophysiology, sMRI, and DTI) were analyzed using each of the nine configurations of machine learning algorithms yielding nine estimates per modality (Fig. 2). In order to compare the contribution of individual variables to these unimodal multivariate estimates, we performed univariate *t* tests between patients and healthy controls (Fig. 3). In order to estimate the multimodal diagnostic accuracy, any modality, which significantly discriminated between patients and healthy controls, was analyzed with all seven combinations in an early integration of the remaining modalities, where variables are concatenated to form larger combined modalities. Finally, we explored if any modality predicted PANSS symptom remission according to the Andreasen criteria (Andreasen *et al*., 2005). Analyses of symptom remission were performed for patients only, and for these analyses, a fifth ‘clinical modality’ was constructed. The clinical modality comprised basic demographic and clinical features, which may influence on illness prognosis: age, gender substance use, DUI, GAF (symptoms and function), and PANSS subscores (positive, negative, and general symptoms). To estimate prediction of symptom remission after 6 weeks of amisulpride treatment, data from each of the five modalities were analyzed using all nine configurations of algorithms via the same analysis pipeline as described above (Fig. 1).

## Results

### Demographics

Forty-six patients and 58 healthy controls were included in the current analyses. Groups were well matched on age, gender, but parental socioeconomic status was lower in patients compared with controls. Compared with controls, the patients had significantly fewer years of education, and significantly higher use of tobacco and recreational drugs, except for use of hallucinogens. Patients were treated with amisulpride in an average dose of 248.4 mg/day for 6 weeks. After 6 weeks of amisulpride treatment, 11 out of 34 (32%) patients fulfilled remission criteria (Andreasen *et al*., 2005) (Table 1).

### Unimodal diagnostic accuracy

Since the two groups differed in size (46 patients and 58 healthy controls), the ‘chance accuracy’ was 56% [(58/(46 + 58) × 100)]. The diagnostic accuracy of cognition ranged between 60% and 69% for all nine configurations of algorithms. A permutation test using 1000 permutations showed that all configurations using cognitive data significantly differentiated between patients and controls (*p* values ranging from 0.001 to 0.009) (see online Supplementary Material, Table S1). The diagnostic accuracy for electrophysiology, sMRI, and DTI ranged between 49% and 56% and did not exceed chance accuracy (Fig. 2).

The planned *t* tests showed that 11/25 of the cognitive variables survived Bonferroni correction (0.05/25 = 0.002) (Fig. 3). The variables covered domains of IQ, working memory, motor function, verbal fluency, processing speed, executive functions, spatial working memory, and sustained attention (see online Supplementary Material, Table S2 for specification of variables). None of 19 electrophysiological, 3/98 sMRI, and 5/100 DTI variables significantly differed between patients and controls at *p* < 0.05; however, none survived after Bonferroni correction (Fig. 3 and online Supplementary Material, Table S2).

### Multimodal diagnostic accuracy

None of the multimodal analyses with cognition plus any combination of one or more of the remaining modalities (electrophysiology, sMRI, and DTI) revealed significantly higher accuracies than cognition alone (accuracies ranging between 51% and 68%) (see online Supplementary Material, Table S1).

### Prognostic ability

Using symptomatic remission (*N* = 11) *v.* non-remission (*N* = 23) as a dichotomous outcome measure equals a ‘chance accuracy’ of 68% [(23/(11 + 23) × 100)]. None of the modalities predicted symptom remission after 6 weeks above chance level: cognition, electrophysiology, sMRI, and DTI predicted symptom remission at accuracies ranging between 48% and 67%. The fifth ‘clinical variable’ predicted symptom remission with accuracies ranging between 51% and 67% (see online Supplementary Material, Table S3).

## Discussion

To our knowledge, this is the first study to investigate the diagnostic accuracy of machine learning algorithms using multimodal data in antipsychotic-naïve, first-episode schizophrenia patients. Contrary to our expectations, we found that only cognitive data, but no other modality, significantly discriminated patients from healthy controls. Moreover, we did not find enhanced accuracies by combining cognition with other modalities, and finally, none of the modalities predicted symptom remission.

Based on cognitive data, all nine configurations of machine learning algorithms could separate patients from healthy controls with a statistically significant accuracy. Supervised machine learning algorithms model the interdependent pattern of variables, which best separate the data with respect to the outcome (e.g. ‘schizophrenia’ or ‘healthy’). Our *t* tests indicated that patients differed from controls on a broad spectrum of cognitive domains, and the feature selection lines shown in Fig. 3*b* indicate that variables with lower *p* values were included more frequently in the machine learning models. Hence, at initial diagnosis of schizophrenia, cognitive deficits appear markedly more pronounced than electrophysiological and neuroanatomical aberrations. Interestingly, two previous multimodal studies in medicated patients also indicated that cognitive parameters yielded higher classification accuracies than sMRI (Karageorgiou *et al*., 2011), and genotype, DTI, and fMRI (Pettersson-Yeo *et al*., 2013). Cognitive deficits are not a part of the diagnostic criteria for schizophrenia, although this has been discussed in the field before the implementation of DSM-5 (Kahn and Keefe, 2013). Our findings support resuming these discussions and examining the evidence for including objective cognitive assessment into future diagnostic systems.

The accuracies regarding neuroanatomical and electrophysiological markers reported in this study are remarkably lower than the accuracies reported in several previous studies. A recent meta-analysis of 20 sMRI studies concluded that application of multivariate algorithms could discriminate schizophrenia patients from healthy controls with a sensitivity of 76% and a specificity of 79% (Kambeitz *et al*., 2015). Higher age and more psychotic symptoms, which in turn may be associated with illness duration and illness severity, more antipsychotic exposure, and more substance abuse, were identified as significant moderators. Moreover, resting-state fMRI data were superior to sMRI in discriminating schizophrenia patients from controls. In the current study, patients were all antipsychotic-naïve, relatively young (mean age of 25.0 years), and displayed moderate psychotic symptoms (PANSS-positive symptoms of 20.1) (Table 1). Furthermore, resting-state fMRI was not included. A previous study using electrophysiological data from 16 schizophrenia patients and 31 healthy controls resulted in a correct classification rate of around 93%. Notably, different EEG measures were used than in the current study, and a mean age of 36 years suggests that the patients were chronically ill and medicated (Santos-Mayo *et al*., 2017). Collectively, the limited clinical confounders in the current study may have contributed to the low diagnostic accuracies of sMRI and DTI, and electrophysiology.

Moreover, methodological differences may contribute to explain the current findings. To optimize the external validity, we applied a rigorous approach in our analysis pipeline. Specifically, we used all available variables, i.e. no feature selection was done prior to entering data into the analysis pipeline. Generally, the studies, which have reported very high accuracies, have first applied a statistical test to pre-select variables, which discriminate between groups on the outcome measure for the specific dataset (e.g. Chu *et al*., 2016; Santos-Mayo *et al*., 2017). A recent SVM study using sMRI cortical thickness and surface data from 163 first-episode, antipsychotic-naïve patients (mean age 23.5 years) and matched controls (mean age 23.6 years) revealed a diagnostic accuracy of 81.8% and 85.0%, respectively, for thickness and surface. In that study, the SVM input comprised variables, which separated patients from controls on a *t* test adjusted for multiple comparisons (Xiao *et al*., 2017). Conversely, a recent machine learning study on voxel-based MRI data from 229 schizophrenia patients and 220 healthy controls from three independent datasets used no prior feature selection and reported low accuracies ranging between 55% and 73.5% (Winterburn *et al*., 2017). Thus, pre-analysis feature selection may provide higher accuracies at the expense of generalizability of the results and should therefore be discouraged in studies aiming at clinical translation.

Contrary to our expectations, we did not find added diagnostic accuracy when combining cognition with other modalities. Moreover, neither cognition nor our constructed ‘clinical variable’ predicted symptom remission after 6 weeks according to criteria which were validated after 6 months of treatment (Andreasen *et al*., 2005). Since the between-subject variability in our data is large, but the group differences between antipsychotic-naïve patients and healthy controls regarding electrophysiology and neuroanatomy are subtle, our results encourage application of multimodal, multivariate analyses in order to disentangle neurobiological distinct subgroups within cohorts of schizophrenia patients. Specifically, multimodal, multivariate analyses may identify clinically meaningful subgroups of schizophrenia patient, e.g. with regard to clinical trajectories (Bak *et al*., 2017). Finally, and in line with the RDoC initiative, it is conceivable that indices of clinical trajectories may expand beyond psychopathology also to encompass more objective, biologically valid assessments.

Some strengths and limitations should be considered. At inclusion, the patients were antipsychotic-naïve and as intervention we used a relatively selective dopamine D_2_ receptor antagonist. Therefore, our diagnostic accuracies reflect minimally confounded estimates of neurobiological disturbances at the earliest stage of schizophrenia. First-episode, antipsychotic-naïve patients are challenging to recruit, and since we required close to complete datasets from all participants on four modalities, the number of included patients may have been too small for optimal modeling of electrophysiology, sMRI, and DTI data. The four modalities used in this study were *a priori* selected because our own eletrophysiological (Düring *et al*., 2014, 2015) and DTI data (Ebdrup *et al*., 2016) as well as abundant independent data have rather consistently shown group differences between schizophrenia patients and controls. Moreover, data on these four modalities can be obtained by means of relatively standardized procedures, which enhances the generalizability our study. As we have also previously published group differences on this cohort in reward processing (Nielsen *et al*., 2012*a*, 2012*b*), resting-state activity (Anhøj *et al*., 2018), and striatal dopamine D_2_ receptor-binding potentials (Wulff *et al*., 2015), inclusion of functional MRI or neurochemical data may have given more positive results. In the current study, we aimed at balancing measures with high clinical generalizability on the largest possible dataset. Because of the absence of standardized pipelines for more dynamic and task-dependent measures, and because inclusion of additional modalities would have reduced the number of participant with full datasets, we *a priori* decided not to include fMRI and neurochemical data in the current analyses. Nevertheless, across all four modalities, our nine different configurations of machine learning algorithms appeared to detect similar signals as the conventional *t* tests (Fig. 3*b*). This overlap in signal provides indirect validation of the applied methods and implies that multivariate algorithms are not a ‘black box’ (Castelvecchi, 2016). As recommended in a recent meta-analysis of machine learning classifications studies, we corrected for age and demographical group differences (Neuhaus and Popescu, 2018). Nevertheless, our modest sample size requires replication in an independent sample, which was currently not available. Regarding prediction of outcome, we only evaluated symptom remission with respect to criteria, which were validated after 6 months of treatment (Andreasen *et al*., 2005). Because our analyses of symptom remission were based on only 34 patients (11 patients were in remission), these results should also be interpreted cautiously since we cannot exclude a Type 2 error.

The inclusion of all available data resulted in an unintended group difference in parental socioeconomic status (Table 1). There were no group differences in premorbid IQ (i.e. DART), but significant group differences on estimated total IQ, with effect sizes similar to previous findings in first-episode samples (Mesholam-Gately *et al*., 2009), but still, these sociodemographic differences cannot explain the marked group differences in cognitive performance we see between groups. We allowed benzodiazepines on an ‘as needed’ basis until 12 h prior to examination days to reduce anxiety and secure sleep. Therefore, we cannot exclude an effect of benzodiazepines on our results; however, since sleep restriction also negatively affects cognition (Lowe *et al*., 2017), we judge the potential bias of benzodiazepines minimal. Our comprehensive approach where we included all available variables may have compromised the signal-to-noise ratio. *A priori* selection of predefined candidate variables, i.e. to make use of ‘domain knowledge’, could potentially have enhanced our signal-to-noise ratio, and in turn our accuracies, without compromising the external validity. Moreover, for neuroanatomical analyses, we included regions of interest. Although a voxel-based approach may be more sensitive to global brain structural aberrations, this was not the case in the recent large machine learning study on voxel-based MRI data mentioned above (Winterburn *et al*., 2017).

Visual inspection of the *t* tests presented in Fig. 3*a* show that the magnitude of cognitive group differences is marked and extensive (22/25 variables had *p* values <0.05), whereas only few variables from electrophysiology, sMRI, and DTI had *p* values <0.05. A more liberal correction for multiple comparisons than the applied Bonferroni correction, e.g. the false discovery rate *ad modum* Benjamini–Hochberg (Benjamini and Hochberg, 1995) would not have changed our overall conclusion that cognitive deficits, compared with electrophysiological and regional brain measures, are core features of schizophrenia at first clinical presentation (Kahn and Keefe, 2013). Since we only investigated one diagnostic category (i.e. schizophrenia), we cannot infer to what extent the discriminative diagnostic patterns of cognitive disturbances are specific to schizophrenia *per se* (Bora and Pantelis, 2016).

In conclusion, this multivariate and multimodal proof-of-concept study on antipsychotic-naïve patients showed that cognition, but not electrophysiological and neuroanatomical data, significantly discriminated schizophrenia patients from healthy controls. Overall, these findings add to the increasing call for cognition to be included in the definition of schizophrenia. To bring about the full potential of machine learning algorithms in first-episode, antipsychotic-naïve schizophrenia patients, careful a *priori* variable selection based on independent data as well as inclusion of other modalities may be required. Machine learning studies aiming at identification of clinically meaningful subgroups of schizophrenia patients are encouraged.

## Supplementary material

For supplementary material accompanying this paper visit https://doi.org/10.1017/S0033291718003781. click here to view supplementary material
