# Supplementary material for: Accuracy of diagnostic classification algorithms using cognitive-, electrophysiological-, and neuroanatomical data in antipsychotic-naïve schizophrenia patients
Source: Psychol Med. 2018 Dec 18;49(16):2754–63. doi: 10.1017/S0033291718003781 (PMC6877469; doi:10.1017/S0033291718003781)
Supplement: Supplementary file 1 [file S0033291718003781sup001.zip › S0033291718003781sup001/Supplementary_Machine_Learning_Text_Ebdrup_2018.pdf]

Supplemental Material

Machine learning algorithms

Below a brief description of the machine learning algorithms and the parameters used in the Matlab pipeline (Figure 1).

Inner loop 5-fold cross validation

- Matlab function: *c=cvpartition(label,'kfold',5)*
- Same cross validation object (*c*) used for all models where needed
- Use of label input stratifies cross validation splits

Backwards feature elimination

- Matlab function: *sequentialfs*
- Parameters:
    - FUN: criterion function based on model in question, e.g. nB
    - cv: c (inner loop cross validation object)
    - Direction backward
- Description:
    - Elimination of features based on their effect on the inner loop validation error
    - Applied to models without any other external or inherent parameter optimization (nB, LR, SVM_l, SVM_h, DT – marked with [w. fs] below)

Configurations of algorithms
nB - Naive Bayes [w. fs]

- Matlab function: *fitcnb*
- No parameter input
- Description:
    - Probabilistic/generative representation of data
    - Each variable is assumed to be conditionally independent from the others given the class
    - Mean and covariance is modeled independently for each feature and each class
    - The independent modeling of covariance for each class can yield non-linear solutions

LR - Logistic regression [w. fs]

- Matlab function: *mnrfit*
- No parameter input
- Description:
    - Sigmoid transformation of the linear combination of variables to provide binary output
    - Linear classifier

LR_r - Logistic regression with L1 regularization

- Matlab function: *lassoglm*
- Parameters:
    - binomial
    - NumLambda: 25
    - CV: c (applies inner loop cross validation object (*c*))

    - Alpha: 1 (pure L1 regularization)
    - MCReps: 1 (in consideration of computational burden)
- Description:
    - Application of L1 regularization to logistic regression forces some weights to become 0, meaning that there is an effective feature selection
    - Choice of regularization parameter lambda is done by the function based on the NumLambda parameter and input data such that largest *lambda* value is estimated to be just sufficient to produce all zero weights
    - Linear classifier

SVM - Support vector machine
- Matlab function: *fitcsvm*
- Applied in three configurations of parameters:
    - SVM_l [w. fs]:
        - KernelFunction: linear
        - BoxConstraint: 1000
    - SVM_h [w. fs]
        - KernelFunction: rbf
        - BoxConstraint: 1000
        - KernelScale: auto
    - SVM_h
        - KernelFunction: rbf
        - Matlab function *bayesopt* used for optimization with parameters:
            - OBJECTIVEFCN: kfoldloss using cross validation object (*c*)
            - BoxConstraint: specified using Matlab function: *optimizableVariable* with parameters:
                - [1e-5,1e5]
                - Transform: log
            - KernelScale: specified using Matlab function: *optimizableVariable* with parameters:
                - [1e-5,1e5]
                - Transform: log
            - IsObjectiveDeterministic: true
            - AcquisitionFunctionName: expected-improvement-plus
- Description:
    - A kernel based classifier, which maximizes the margin between the decision hyper plane and any of the observations
    - BoxConstraint is penalty for false positives (called C in literature)
    - Can be made non-linear through the choice of kernel e.g. rbf kernel, where the kernel width determines magnitude of non-linearity and is a parameter that must be set or optimized

DT - Decision tree [w. fs]
- Matlab function: *fitctree*
- No parameter input
- Description:
    - Classification based on several thresholds on individual or multiple variables
    - Different classification rules apply to different areas of input space, i.e. the tree is not necessarily symmetric
    - Non-linear classifier

RF - Random forests

- Matlab function: TreeBagger
- Parameters:
    - NUMTREES: 500
- Description:
    - Tree based boosting method where many (here 500) different trees are modeled on random subsets of variables and observations, which overlap.
    - All trees are trained individually on the task and then averaged in the final decision-making
    - Non-linear classifier
