# Supplementary material for: Accuracy of diagnostic classification algorithms using cognitive-, electrophysiological-, and neuroanatomical data in antipsychotic-naïve schizophrenia patients
Source: Psychol Med. 2018 Dec 18;49(16):2754–63. doi: 10.1017/S0033291718003781 (PMC6877469; doi:10.1017/S0033291718003781)
Supplement: Supplementary file 1 [file S0033291718003781sup001.zip › S0033291718003781sup001/Supplementary_Table S1_Ebdrup_2018.pdf]

Supplementary Table S1

Table S1 shows the diagnostic accuracies for all nine configurations of algorithms for unimodal and multimodal analyses. * Only cognition underwent permutation tests to evaluate if accuracies were significantly above chance level (56%). For cognition, all accuracies for all nine configurations of alorithms were significant. nB: p = 0.002. LR: p = 0.009. LR_r: p = 0.001. SVM_l: p = 0.004. SVM_h: p = 0.002. SVM_o: p = 0.001. DT: p = 0.002. RF: p = 0.002. AS: p = 0.001.

	**nB**	**LR**	**LR_r**	**SVM_l**	**SVM_h**	**SVM_o**	**DT**	**RF**	**AS**
**Cog***	0.68	0.61	0.69	0.60	0.64	0.67	0.61	0.69	0.67
**EEG**	0.53	0.53	0.55	0.55	0.52	0.54	0.55	0.56	0.53
**sMRI**	0.51	0.50	0.54	0.52	0.53	0.53	0.49	0.51	0.51
**DTI**	0.53	0.53	0.55	0.54	0.53	0.54	0.52	0.53	0.51
**Cog EEG**	0.67	0.58	0.68	0.57	0.62	0.64	0.58	0.68	0.64
**Cog sMRI**	0.66	0.53	0.64	0.60	0.62	0.63	0.56	0.66	0.63
**Cog DTI**	0.64	0.52	0.67	0.56	0.65	0.63	0.59	0.65	0.64
**Cog EEG sMRI**	0.64	0.54	0.64	0.58	0.61	0.62	0.57	0.66	0.62
**Cog EEG DTI**	0.62	0.53	0.66	0.54	0.61	0.61	0.58	0.65	0.62
**Cog sMRI DTI**	0.63	0.51	0.63	0.57	0.61	0.60	0.56	0.64	0.62
**Cog EEG sMRI DTI**	0.62	0.52	0.63	0.56	0.60	0.60	0.56	0.64	0.61
