# Supplementary material for: Accuracy of diagnostic classification algorithms using cognitive-, electrophysiological-, and neuroanatomical data in antipsychotic-naïve schizophrenia patients
Source: Psychol Med. 2018 Dec 18;49(16):2754–63. doi: 10.1017/S0033291718003781 (PMC6877469; doi:10.1017/S0033291718003781)
Supplement: Supplementary file 1 [file S0033291718003781sup001.zip › S0033291718003781sup001/Supplementary_Table_S2_Ebdrup_2018.pdf]

Supplementary Table S2

Cognition	Electrophysiology	Structural MRI	Diffusion Tensor Imaging
		48 region names below, values for left and right. 96 features in total	20 tracts, 5 (normalized) values for each (MO,MD,L2/L3, L1, FA), 100 features in total
Premorbid IQ (DART)	P50 c-stimulus Amplitude	Frontal Pole, L, R	Right uncinate fasciculus, MO,MD,L2/L3, L1, FA
**Verbal IQ a ***	P50 t-stimulus Amplitude	Insular Cortex, L, R	Left uncinate fasciculus, MO,MD,L2/L3, L1, FA
**Performance IQ a ***	P50 t/c ratio	Superior Frontal Gyrus, L, R	Right superior longitudinal fasciculus temporal part, MO,MD,L2/L3, L1, FA
**Full scale IQ a ****	P50 c-stimulus Latency	Middle Frontal Gyrus, L, R	Left superior longitudinal fasciculus temporal part, MO,MD,L2/L3, L1, FA
**List learning b ***	PPI 85dB 120ms	Inferior Frontal Gyrus pars triangularis, L, R	Right superior longitudinal fasciculus, MO,MD,L2/L3, L1, FA
**Digit Sequencing Task b ****	PPI 85dB 60ms	**Inferior Frontal Gyrus pars opercularis, L*, R**	Left superior longitudinal fasciculus, MO,MD,L2/L3, L1, FA
**Token Motor Task b ****	PPI 76dB 120ms	Precentral Gyrus, L, R	**Right inferior longitudinal fasciculus, MO,MD,L2/L3, L1, FA***
**Verbal Fluency ("supermarket") b ****	PPI 76dB 60ms	Temporal Pole, L, R	**Left inferior longitudinal fasciculus, MO,MD,L2/L3, L1, FA***
**Verbal Fluency ("F") b ****	PPI Pulse alone	Superior Tempora lGyrus Anterior Division, L, R	**Right inferior fronto occipital fasciculus, MO,MD,L2/L3, L1, FA***
**Verbal Fluency ("S") b ***	PPI 85dB 120ms amplitude	Superior Temporal Gyrus Posterior Division, L, R	Left inferior fronto occipital fasciculus, MO,MD,L2/L3, L1, FA
**Symbol coding b ****	PPI 85dB 60ms Amplitude	Middle Temporal Gyrus Anterior Divivision, L, R	Forceps minor, MO,MD,L2/L3, L1, FA
**Tower of London b ****	PPI 76dB 120ms Amplitude	Middle Temporal Gyrus Posterior Division, L, R	Forceps major, MO,MD,L2/L3, L1, FA
**Spatial Span c ***	PPI 76dB 60ms Amplitude	Middle Temporal Gyrus temporo occipital part, L, R	Right corticospinal tract, MO,MD,L2/L3, L1, FA
**Spatial Working Memory (strategy) c ****	MMN Frequency deviant FCZ	Inferior Temporal Gyrus Anterior Division, L, R	Left corticospinal tract, MO,MD,L2/L3, L1, FA
**Spatial Working Memory (total errors) c ****	MMN Duration deviant FCZ	Inferior Tempora lGyrus Posterior Division, L, R	Right cingulum hippocampus, MO,MD,L2/L3, L1, FA
**Stockings of Cambridge (problems solved) c ***	MMN Frequency and duration deviant FCZ	Inferior Temporal Gyrus temporo occipital part, L, R	**Left cingulum hippocampus, MO,MD,L2/L3, L1, FA***
Stockings of Cambridge (initial thinking time) c	MMN Frequency deviant Latency	Postcentral Gyrus, L, R	Right cingulum cingulate gyrus, MO,MD,L2/L3, L1, FA
**Intra-Extra Dimensional Set Shift (errors) c ****	MMN Duration deviant Latency	Superior Parietal Lobule, L, R	**Left cingulum cingulate gyrus, MO,MD,L2/L3, L1, FA***
**Intra-Extra Dimensional Set Shift (EDS errors) c ***	MMN Frequency and Duration deviant Latency	Supramarginal Gyrus Anterior Division, L, R	Right anterior thalamic radiation, MO,MD,L2/L3, L1, FA
**Reaction Time (simple reaction) c ***		Supramarginal Gyrus Posterior Division, L, R	Left anterior thalamic radiation, MO,MD,L2/L3, L1, FA
Reaction Time (simple movement) c		Angular Gyrus, L, R	
**Reaction Time (choice reaction) c ***		**Lateral Occipital Cortex superior Division, L*, R**	
Reaction Time (choice movement) c		Lateral Occipital Cortex inferior Division, L, R	
**Rapid Visual Processing (A´, 3-5-7) c ****		Intracalcarine Cortex, L, R	
**Rapid Visual Processing (A´, 3-5-7, 2-4-6) c ***		Frontal Medial Cortex, L, R	
		Supplementary Motor Cortex, L, R	
		Subcallosal Cortex, L, R	
		Paracingulate Gyrus, L, R	
		Cingulate Gyrus Anterior Division, L, R	
		Cingulate Gyrus Posterior Division, L, R	
		Precuneous Cortex, L, R	
		Cuneal Cortex, L, R	
		Frontal Orbital Cortex, L, R	
		Parahippocampal Gyrus Anterior Division, L, R	
		Parahippocampal Gyrus Posterior Division, L, R	
		Lingual Gyrus, L, R	
		Temporal Fusiform Cortex Anterior Division, L, R	
		Temporal Fusiform Cortex Posterior Division, L, R	
		Temporal Occipital Fusiform Cortex, L, R	
		Occipital Fusiform Gyrus, L, R	
		Frontal Operculum Cortex, L, R	
		Central Opercular Cortex, L, R	
		Parietal Operculum Cortex, L, R	
		Planum Polare, L, R	
		Heschls Gyrus, L, R	
		Planum Temporale, L, R	
		**Supracalcarine Cortex, L*, R**	
		Occipital Pole, L, R	

*Table S2 shows all variables included in the four modalities (see Figure 3). Unimodal significant differences between patients and controls are shown in bold. * indicates p<0.05. ** indicate p-value significant after Bonferroni correction. [a] WAIS III. [b] CANTAB. [c] BACS. P50 = P50 suppression. PPI = Pre-Pulse Inhibition of the startle reflex. MMN = Mismatch Negativity. L = left. R = Right. MO = Mode of anisotropy. MD = Mean diffusivity. L2/L3 = Radial diffusivity (λ23). L1 = Parallel diffusivity (λ1). FA = Fractional Anisotropy.*
