# Supplementary material for: Accuracy of diagnostic classification algorithms using cognitive-, electrophysiological-, and neuroanatomical data in antipsychotic-naïve schizophrenia patients
Source: Psychol Med. 2018 Dec 18;49(16):2754–63. doi: 10.1017/S0033291718003781 (PMC6877469; doi:10.1017/S0033291718003781)
Supplement: Supplementary file 1 [file S0033291718003781sup001.zip › S0033291718003781sup001/Supplementary_Table_S3_Ebdrup_2018.pdf]

Supplementary Table S3

Table S3 shows the unimodal prognostic accuracies (i.e. symptom remission estimates) for all nine configurations algorithms. No accuracies were above chance level (68%).

	nB	LR	LR_r	SVM_l	SVM_h	SVM_o	DT	RF	AS
**Cog**	0.59	0.51	0.62	0.57	0.64	0.64	0.48	0.56	0.53
**EEG**	0.48	0.48	0.66	0.56	0.58	0.64	0.50	0.49	0.51
**sMRI**	0.61	0.51	0.67	0.63	0.60	0.64	0.63	0.67	0.54
**DTI**	0.55	0.50	0.66	0.60	0.61	0.63	0.52	0.65	0.57
**Clinical modality**	0.67	0.51	0.62	0.60	0.62	0.60	0.56	0.64	0.55
